# Narrative Style Influences Citation Frequency in Climate Change Science

**DOI:** 10.1371/journal.pone.0167983

**Published:** 2016-12-15

**Authors:** Ann Hillier, Ryan P. Kelly, Terrie Klinger

**Affiliations:** School of Marine & Environmental Affairs, University of Washington, Seattle, Washington, United States of America; University of Brighton, UNITED KINGDOM

## Abstract

Peer-reviewed publications focusing on climate change are growing exponentially with the consequence that the uptake and influence of individual papers varies greatly. Here, we derive metrics of narrativity from psychology and literary theory, and use these metrics to test the hypothesis that more narrative climate change writing is more likely to be influential, using citation frequency as a proxy for influence. From a sample of 732 scientific abstracts drawn from the climate change literature, we find that articles with more narrative abstracts are cited more often. This effect is closely associated with journal identity: higher-impact journals tend to feature more narrative articles, and these articles tend to be cited more often. These results suggest that writing in a more narrative style increases the uptake and influence of articles in climate literature, and perhaps in scientific literature more broadly.

## Introduction

Climate change is among the most compelling issues now confronting science and society, and climate science as a research endeavor has grown accordingly over the past decade. The number of scholarly publications is increasing exponentially, doubling every 5–6 years [1]. The volume of climate science publications now being produced far exceeds the ability of individual investigators to read, remember, and use. Accordingly, it is increasingly important that individual articles be presented in a way that facilitates the uptake of climate science and increases the salience of their individual research contributions.

Evidence from psychology and literary theory suggests that audiences better understand and remember narrative writing in comparison with expository writing [2,3], and new evidence from neuroscience has revealed a specific region in the brain that is activated by stories [4]. Narrative writing tells a story through related events [5], whereas expository writing relates facts without much social context. Presenting the same information in a more narrative way has the potential to increase its uptake—an especially attractive prospect in the context of climate science and scientific writing generally—and consequently, narratives are widely recognized as powerful tools of communication [2,6].

Despite this, professional scientific writing tends to be more expository than narrative, prioritizing objective observations made by detached researchers and relying on the logical proposition “if X, then Y” to define the structure of the argument [7]. Narrative writing, on the other hand, is commonly used to good effect in popular science writing [8]. Both simple narratives and apocalyptic climate narratives are known to capture public attention and spur action [9–11]. Moreover, narratives can influence perceptions of climate risk and policy preferences among the public [12], and the narrative style has been proposed as a powerful means of research to address problems of knowledge, policy, and action as they relate to climate change [13].

Here we explore the influence of narrative in the professional communication of climate science research, acknowledging that the perception of narrative can be subjective and context-dependent [14,15]. We hypothesized that scientific papers with more narrative text are more likely to be highly cited than those with less narrative (i.e., more expository) text, using citation frequency as a proxy for a paper’s influence on the field at large. To test this hypothesis, we derived six elements of narrativity from studies on narrative comprehension [15–17] and the literatures of psychology [2,18,19] and narrative theory [14,20,21], and used these six elements to evaluate the degree of narrativity in 732 abstracts taken from the peer-reviewed scientific literature on climate change. We then assessed the relationship between narrativity in these journal abstracts in the context of other factors known to influence citation rate, including journal identity, abstract length, and number of authors.

## Methods

### Abstract Selection

We analyzed abstracts instead of the full text of selected papers because the abstract typically is the first section of the paper viewed by readers; moreover, the abstract is the only section of the paper immediately available on databases such as PubMed [22]. Hence, abstracts provide a relatively consistent point of entry to scientific publications. To select focal abstracts for the dataset, we first used the PubMed database to select the journals that published the largest number of articles featuring the phrase “climate change” in the abstract or title between 2009 and 2010. Our reasoning for choosing the set of papers that we did was as follows: First, we limited the scope by the field of inquiry (climate change), hoping to minimize the statistical variance (or “noise”) that would probably have resulted from an analysis that included many fields (which in turn likely differ in citation frequencies and writing conventions, among other relevant factors). Next, we reasoned that it takes a number of years for papers to accrue a number of citations—and consequently for a set of papers to develop a distribution of citation counts—that would allow us to test our core hypothesis. We began this study in 2015, and chose 5-to-6 years as a reasonable window, allowing for citations to accrue, but not letting the papers become outdated. Finally, knowing that citations accrue to individual papers nonlinearly over time, we recognized the difficulty in using the available data (total citations, rather than citations-by-year for each paper) to derive time-correction factors for each paper in the dataset. Consequently, we featured only papers from a narrow time window, minimizing the effect of time-since-publication on the distribution of citations in our dataset.

We identified 19 journals with the largest number of articles meeting these criteria, and then retrieved the abstracts, citation counts, and other relevant information through the database Web of Science (S1 Table; raw dataset N = 802 abstracts; N = 732 after quality control; see below). These abstracts differed in citation frequency by two orders of magnitude, having been cited between 1 and 1205 times as of March 30, 2016 (median = 69; we did not collect data on papers with zero citations in order to avoid the problems associated with log-transforming zero data), and reflected the expected left-skewed distribution.

### Crowdsourcing

We used the crowdsourcing site CrowdFlower (http://www.crowdflower.com) to collect information regarding the narrativity of each abstract. Crowdsourcing—in which many individuals are paid small amounts of money to complete discrete parts of a much larger task—as a research method is growing as technical capacity increases [23]. It offers an efficient research tool for work that requires a degree of human assessment spread over a large number of data points, with access to a diverse, skilled workforce, and produces reliable data in comparison with alternative methods [24,25].

The CrowdFlower platform allowed us to: 1) collect reader-coded information for a large number of abstracts that could not be collected by text-mining or other means; 2) collect multiple (n = 7) independent assessments (“judgments”) about the narrativity of each abstract; and simultaneously 3) include human interpretation and discretion in the quantification of narrativity. We collected multiple judgments for each abstract as a means of quality-control, given that individual readers can perceive narrativity somewhat differently [26].

Online contributors evaluated abstracts by first reading instructions (S1 Text) and an example question, and then answering a series of six questions (S2 Text) for each abstract. These questions were intended to evaluate each abstract with respect to indicators of narrativity (described in the next section). Contributors were paid per submitted page, each of which included five abstracts and the corresponding questions.

We used the following measures to ensure high quality responses: 1) gave access to this job only to CrowdFlower’s highest ranked contributors (the site ranks them based upon past performance); 2) set a minimum completion time for each page of work; and 3) restricted contributor location to a number of countries in which English is the primary language and literacy rates are high: Australia, Canada, New Zealand, United Kingdom, and United States. Although our primary reason for imposing this restriction was based on language skills, we note that these countries largely correspond to those that dominate climate change publications, both in terms of number and citation frequency [1]. A total of 155 individual contributors evaluated the abstracts used in this study.

### Independent Variables: Narrative Indicators

To derive indicators of narrativity, we adapted methods and indicators based on comparable studies [15–17] and supported by relevant literature from narrative theory [14,20,21], psychology [2,18,19], communications [27], philosophy [28], and history [26]. We chose indicators to reflect *setting*, *narrative perspective*, *sensory language*, *conjunctions*, *connectivity*, and *appeal*.

*Setting* provides a description of where and when events take place and is of the fundamental components of narratives. The spatial and temporal dimensions established by setting help create a mental image that distinguishes narratives from other forms of discourse [20]. We assessed setting by asking contributors whether there is a specific mention of place or time in the abstract [16].

*Narrative perspective* describes the position or role of the narrator. According to Lejano et al. [15], the presence of a narrator distinguishes narratives from other forms of communication—that is, narrators tell narratives. The narrator is responsible for eliciting emotions in the reader [29]. First-person narrators have a stronger narrative presence than other narrative perspectives, such as third-person or no narrator [2,16]. We assessed narrative perspective by asking contributors whether or not the narrator referred to himself in the text (e.g., through use of pronouns such as I, we, and our).

*Sensory language* appeals to the senses and emotions of the reader and can be used to establish personal identity, for example, through the narrator expressing “emotions, attitudes, beliefs, and interpretations” [20]. Accordingly, we assessed sensory language by asking contributors to count the number of times that sensory or emotional language appeared in the abstract. We then normalized the resulting counts by abstract length (number of words).

*Conjunctions* are used to connect words and phrases, binding narratives together in a logical form [17]. We used the presence of conjunctions to determine the extent to which an abstract is logically ordered, based on the observation that a temporal or causal ordering of events is an essential, and distinguishing, characteristic of narratives [15,30–33], one which implies momentum towards completion [20] and evokes human understanding [21]. We assessed the use of conjunctions by asking contributors to count the number of times that conjunctions signifying cause and effect, contrast, or temporal ordering appeared in the text. We then normalized the resulting counts by abstract length.

*Connectivity* refers to words or phrases that create explicit links within the text, either as a specific reference back to the same thing or repetition of a word from the previous sentence, provided it carries the same meaning [17]. We assessed connectivity by asking contributors to count the number of times that words or phrases from one sentence were used to create an explicit link to the sentence immediately preceding it. We provided contributors the additional instruction to look for logical linkage between ideas. We then normalized the resulting counts by abstract length.

*Appeal* refers to the moral or evaluative orientation of a narrative [22]. Appeal in the form of evaluative commentary or ‘landscape of consciousness’ is an important aspect of narrativity [14,21], answering the question of *why* the story is being told. We assessed the use of appeal by asking contributors if the text makes an explicit appeal to the reader or a clear recommendation for action [16].

### Independent Variables: Other

In addition to the crowdsourced assessments of narrative elements, we collected information on length of abstract (number of words), number of authors, year of publication, journal identity, and journal impact factor. These factors are known to influence the citation rate of peer-reviewed literature [34–36] and were available via Web of Science for each abstract in the dataset.

### Dependent Variable: Citation Frequency

We used citation frequency as a measure of article influence. A large body of literature supports the use of citation analyses as frameworks for evaluating science communication [34,36–38]. Citations reflect the cumulative nature of science and the extent to which a piece of work is represented in a body of literature [36], and can therefore be used as to evaluate the degree of influence of a publication on its field. We used Web of Science to establish the number of citations for the articles associated with each abstract in our dataset. We log-transformed citation counts to account for the skewed distribution in citations.

### Quality Control

We treated Question 2, “Does the narrator refer to himself in the text?” as a “test” question, or secondary quality-control mechanism, due to its objectivity (i.e., unlike some of the other narrative indicators, the existence of a first-person narrator has a “true” answer). After considering all seven responses for this question, respondents who answered in the majority were included in the analysis, whereas respondents who answered in the minority were assumed to be in error and their responses were omitted entirely from the analysis. This improved our confidence in the responses and subsequent analysis. After omitting these minority responses, we averaged the scores across remaining responses for each independent variable to yield a dataset with one value per indicator for each abstract.

Narrative variables with “yes/no” categorical responses (i.e., the indicators “setting”, “narrative perspective”, and “appeal”) were assigned numeric binary values (0 or 1) by rounding respondents’ mean scores (e.g., where 5 out of 7 respondents scored an abstract as having a direct appeal to the reader, the mean appeal score for the abstract was 5/7, or 0.71, and we rounded this score to 1 to reflect the idea that the abstract did indeed contain a direct appeal). We used the mean response scores for the other, non-binary narrative variables (“conjunctions”, “connectivity”, and “sensory”). This turned an otherwise discrete variable into a continuous variable, creating an index that captured variations in perceptions of narrativity. For example, contributors might count different numbers of connective phrases and links in a piece of text. Taking the mean, and thereby including the disagreement among responses, produced an overall measure of perceived connectivity for that piece of text. These methods incorporated the subjective nature of narrativity into the results.

### Analysis

Three of our narrative elements were binary, and we therefore used a Wilcoxon Rank-Sum test to test for an association between the presence of these elements and a change in citation frequency. The remaining three narrative elements were continuous variables with non-normal distributions (Shapiro-Wilk test; p < 0.001), and accordingly we used the nonparametric Spearman’s rho to test for correlations between these elements and citation frequency.

In order to account for co-linearities among our narrative elements, we used a principal components analysis to create a single index of narrativity. PC1 alone explained 76.5% of the variance in the narrative elements, with PC2 explaining an additional 13.8%. PCA loadings are given in S2 Table. All analyses were carried out in R [39], and the analysis script and raw dataset are available in supporting files. We also analyzed a version of the same dataset omitting extreme values in both dependent and independent variables (S1 Fig) obtaining nearly identical results as we report here for the full dataset.

Finally, we used simple and multiple linear regression to test for significant associations between groups of variables and citations, and to illustrate the correlation between our narrative index (PC1) and journal impact factor.

## Results

### Individual Indicators of Narrativity

**Fig 1:**
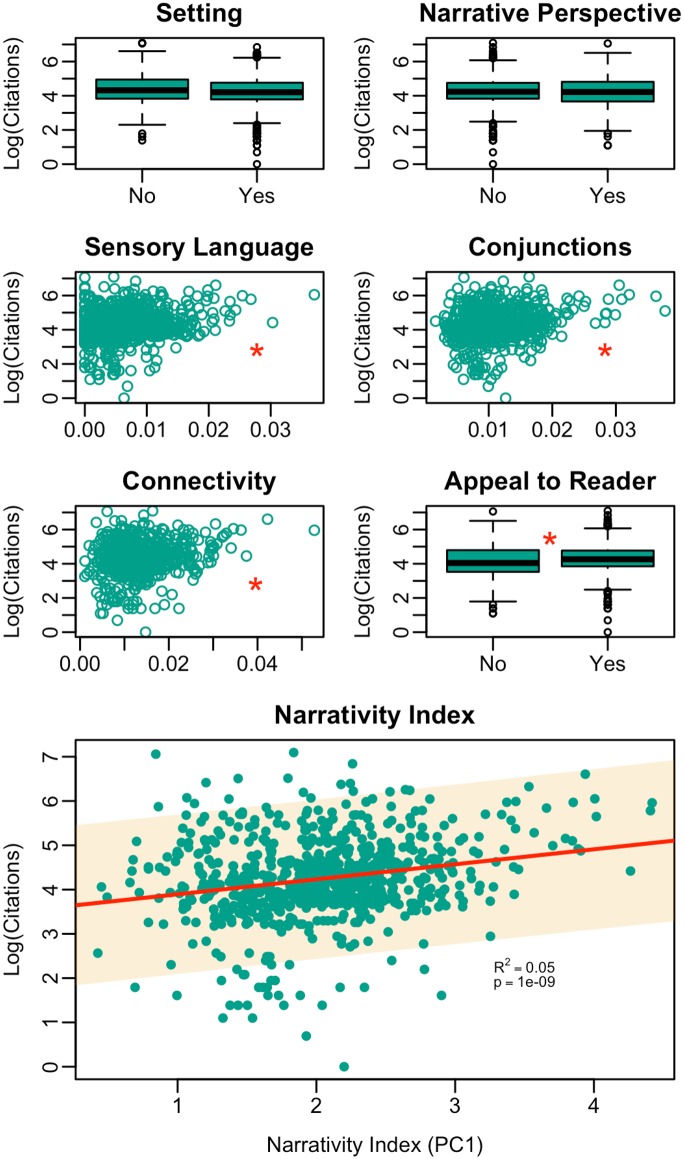
Multipanel plot depicting the relationship between narrativity (individual indicators and single narrativity index given by PC1, labeled individually) and article citation frequency. The use of sensory language, conjunctions, connectivity, and appeal to the reader are significantly correlated with article citation frequency. PC1 index of narrativity is significantly correlated with article citation frequency (linear regression; shaded area indicates 95% confidence interval for the linear model parameters).

Four of six narrative elements were positively associated with article citation frequency (Fig 1). We obtained similar results when holding the year of publication constant (S2 and S3 Figs) and when analyzing the same dataset with outliers excluded (S1 Fig), indicating that neither publication year nor extreme data points substantially affect the trends we report here. Table 1 shows p-values for nonparametric tests (Wilcoxon Rank Sum for binary variables; Spearman correlations for continuous variables), and gives Spearman’s rho for continuous variables.

**Table 1:** Nonparametric relationships between each narrative element and log(citations). For continuous variables, spearman correlations are given along with associated p-values. For binary variables, p-values are given for Wilcoxon rank-sum tests.

	Rho	p-value
Setting	-	0.36
Narrative Perspective	-	0.32
**Sensory**	**0.138**	**1.7x10**^**-4**^
**Conjunctions**	**0.211**	**7.9x10**^**-9**^
**Connectivity**	**0.171**	**3.3x10**^**-6**^
**Appeal**	-	**3.7x10**^**-3**^

**Fig 2:**
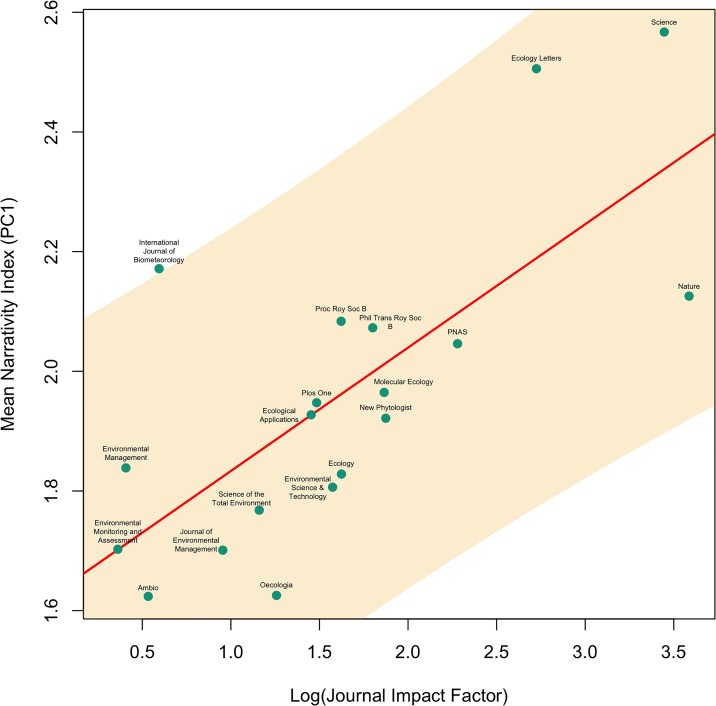
The relationship between the narrativity index (PC1) and journal impact factor. Response variables reflect journal means for articles in our dataset (N = 732); shaded area represents the 95% confidence interval for the best-fit line. Linear regression R^2^ = 0.62, p = 6 x 10^−5^.

Following ordination of the six narrative elements using PCA, PC1 served as our index of narrativity, and was significantly correlated with log(citations) (R^2^ = 0.05, p = 10^−9^; Fig 1). PC1 (Narrativity index) varied significantly among journals (p = 10^−15^), and correlated strongly and positively with log journal impact factor (R^2^ = 0.62, p = 6 x 10^−5^; carried out on PC1 journal means to avoid pseudoreplication), such that higher-impact journals tended to have more narrative articles (Fig 2).

### Non-narrative Independent Variables

We found no citation effect for abstract length after accounting for journal (different journals feature abstracts of different lengths); papers with more authors had subtly, but significantly, more citations than those with fewer authors even after controlling for journal (log(N authors), p < 10^−6^; each additional author was associated with an additional 0.4 citations in the dataset). Citations varied predictably by journal (ANOVA; R^2^ = 0.43; p < 10^−15^), and this effect was largely captured by journal impact factor (R^2^ = 0.37; p < 10^−15^; log(2010 impact factor)). Year of publication (2009 vs. 2010) had a small but significant effect on citations (R^2^ = 0.05; p = 10^−10^; the average paper from 2010 had 1.5 fewer citations than the average paper from 2009).

### Multiple Linear Regression

The best multiple linear regression model included Year, PC1 (narrativity index), (log) Number of Authors, and (log) Impact Factor as independent variables following stepwise model selection using AIC. Taken together, these variables explained 41% of the variance in citations for our dataset (p < 10^−15^).

## Discussion

Our results reveal that—at least among the set of peer-reviewed climate change literature included in our dataset—articles featuring more narrative writing styles are more often cited. This effect is independent of year of publication, number of authors, or abstract length. Of the narrative elements we tested for, the use of sensory language, conjunctions, connectivity between sentences, and appeal (or plea) to the reader all positively and significantly influenced citation frequency. Of these four attributes, appeal [i.e., to the reader] is perhaps most broadly construed and least understood. Nevertheless, the fact that appeal emerged as a key factor in the PCA suggests its importance in climate science writing. It could be the case that appeal is positively associated with narrativity because, in the context of climate science, authors are likely to offer a recommendation (where recommendation is one definition of the term) that is identifiable to or understood by the reader.

Our findings are consistent with the prevailing understanding across a range of fields that audiences tend to understand and recall narratives—that is, stories—far better than information received in other ways [2,14,18–21]. The result is surprising, though, in the context of professional scientific communication, in which expository styles dominate the published literature, word counts are strictly limited by editorial policies, graphics are routinely used to present results, and citation frequency is often considered to depend largely—even primarily—upon the strength of the science. These conventions and constraints would seem to eliminate any role for narrativity in professional scientific writing, but our results indicate otherwise.

Despite the significant effect of narrative style, we found the journal of publication—particularly as captured by the journal’s impact factor—was most closely associated with citation frequency of individual articles. However, we found an unexpectedly strong correlation between narrativity and journal impact factor: more highly cited journals feature more narrative writing styles. We might speculate that this effect stems from differences in editorial policies that subtly encourage or discourage narrative styles, or that, especially in the case of *Nature* and *Science*, effectively communicating to a highly interdisciplinary audience requires a more narrative style. It may also be that more senior authors—presumably publishing in higher-impact journals more often—feel freer to write in a more narrative style. Whatever the reason, the message to authors is clear: up to a point, more narrative writing styles can increase the uptake and ultimate visibility of one’s research.

Our study design did not allow us to test the mechanism(s) of association between narrativity and citation frequency. However, our results add to a growing literature that underscores an important role for narrative communication structure in readers’ abilities to process and recall information. Without knowing the specific cognitive mechanism(s) involved, it appears that the uptake and subsequent use of scientific information is positively influenced by narrative writing styles.

Peer-reviewed scientific discourse is often viewed as a special form of communication, exempt from the qualities of narratives that humans inherently relate to. However, our findings support an alternative interpretation: scientists can engage readers and increase uptake by incorporating narrative attributes into their writing styles. Among the variables we tested, connectedness, or the extent to which sentences are logically related, has the greatest positive influence. Moreover, the use of evaluative commentary can be used to positive effect. By incorporating such attributes into their writing, scientists can more closely mirror the way we as humans experience and understand the world.

## Supporting Information

S1 Table Source journals included in this study. (DOCX)

S2 Table Summary of principal components analysis of narrative elements. (DOCX)

S1 Text CrowdFlower job instructions. (DOCX)

S2 Text CrowdFlower job questions. (DOCX)

S1 Fig Multipanel plot depicting the relationship between narrativity (single variables and composite index panels, as labeled) and log article citation frequency with the 46 outlier abstracts removed. We identified outlier abstracts by fitting appropriate probability distributions to the non-binary independent variables (“conjunctions” (per abstract word), “connectivity” (per abstract word), log(abstract length), log(number of authors); gamma, gamma, normal, and gamma distributions, respectively) and to the dependent variable (log(citations); normal), and excluding responses with a likelihood < 0.01. Consequently, abstracts with very large or very small numbers of conjunctions or connective phrases—or extreme values for word count, number of authors, or number of citations—were removed from the dataset. In total, 46 outliers were removed from the dataset. This figure shows the results of the analyses described in the main paper, but carried out on this dataset with the 46 outlier abstracts removed.
(TIFF)

S2 Fig Multipanel plot depicting the relationship between narrativity (single variables and composite index panels, as labeled) and log article citation frequency for publication year 2009. (TIFF)

S3 Fig Multipanel plot depicting the relationship between narrativity (single variables and composite index panels, as labeled) and log article citation frequency for publication year 2010. (TIFF)

S1 Script R script used in the analysis. (R)

S1 Dataset Raw dataset used in the analysis. (CSV)

S1 Impact Journal dataset used in the analysis. (CSV)
