# Supplementary material for: Narrative Style Influences Citation Frequency in Climate Change Science
Source: PLoS One. 2016 Dec 15;11(12):e0167983. doi: 10.1371/journal.pone.0167983 (PMC5158318; doi:10.1371/journal.pone.0167983)
Supplement: S1 Table — (DOCX) [file pone.0167983.s001.docx]

S1 Table. Source journals included in this study.

| **Journal** |
| --- |
| 1.Ambio |
| 2. Ecological Applications |
| 3. Ecology |
| 4. Ecology Letters |
| 5. Environmental Management |
| 6. Environmental Monitoring and Assessment |
| 7. Environmental Science & Technology |
| 8. International Journal of Biometeorology |
| 9. Journal of Environmental Management |
| 10. Molecular Ecology |
| 11. Nature |
| 12. Oecologia |
| 13. Philosophical Transactions of the Royal Society of London. Series B, Biological Sciences |
| 14. PLoS One |
| 15. Proceedings of the National Academy of Science of the United States of America |
| 16. Proceedings of the Royal Society of London, Series B, Biological Sciences |
| 17. Science |
| 18. The New Phytologist |
| 19. The Science of the Total Environment |
