# Supplementary material for: Narrative Style Influences Citation Frequency in Climate Change Science
Source: PLoS One. 2016 Dec 15;11(12):e0167983. doi: 10.1371/journal.pone.0167983 (PMC5158318; doi:10.1371/journal.pone.0167983)
Supplement: S2 Table — (DOCX) [file pone.0167983.s002.docx]

S2 Table. Summary of Principal Component Analysis of Narrative Elements.

| **Narrative Element** | **PC1** | **PC2** | **PC3** | **PC4** | **PC5** | **PC6** |
| --- | --- | --- | --- | --- | --- | --- |
| Setting | -0.425 | -0.039 | -0.608 | -0.601 | 0.295 | -0.011 |
| Narrative Perspective | -0.27 | 0.885 | -0.064 | 0.019 | -0.369 | -0.048 |
| Sensory | -0.414 | -0.395 | 0.285 | -0.305 | -0.691 | -0.143 |
| Conjunctions | -0.448 | 0.039 | 0.407 | 0.012 | 0.252 | 0.754 |
| Connectivity | -0.446 | 0.048 | 0.393 | 0.155 | 0.464 | -0.637 |
| Appeal | -0.419 | -0.233 | -0.475 | 0.722 | -0.141 | 0.055 |
|  |  |  |  |  |  |  |
|  |  |  |  |  |  |  |
|  |  |  |  |  |  |  |
|  | **PC1** | **PC2** | **PC3** | **PC4** | **PC5** | **PC6** |
| Standard deviation | 2.142 | 0.911 | 0.522 | 0.405 | 0.309 | 0.221 |
| Proportion of Variance | 0.765 | 0.138 | 0.045 | 0.027 | 0.016 | 0.008 |
| Cumulative Proportion | 0.765 | 0.903 | 0.949 | 0.976 | 0.992 | 1 |
