# Supplementary material for: Narrative Style Influences Citation Frequency in Climate Change Science
Source: PLoS One. 2016 Dec 15;11(12):e0167983. doi: 10.1371/journal.pone.0167983 (PMC5158318; doi:10.1371/journal.pone.0167983)
Supplement: S1 Text — (DOCX) [file pone.0167983.s003.docx]

S1 Text. CrowdFlower job instructions

*Identify Attributes of Scientific Writing*

### *Overview*

In this job, you will be presented with a piece of text from a scientific article. After reading the text, you will be asked to identify various attributes of scientific writing. Your input will be used to help form a better understanding of science communication.

### *Process*

Read the text and answer each question. You may wish to review the questions before reading the text for the first time.

*Do:*

Read each question carefully. For each question, read the text at least once. Refer to the text as often as necessary. For questions that require you to count, you may wish to have a pencil and paper handy to keep running tallies.

*Do not:*

Do not read the text only once. Please do not randomly guess answers!

*Summary:*

You will read the text and identify the attributes that the questions describe. Pay close attention to the instructions for each question, and refer to the text as often as necessary.

### *Thank You!*

Your input is valuable to us. Thank you for taking the time to complete this job!
