# Supplementary material for: Narrative Style Influences Citation Frequency in Climate Change Science
Source: PLoS One. 2016 Dec 15;11(12):e0167983. doi: 10.1371/journal.pone.0167983 (PMC5158318; doi:10.1371/journal.pone.0167983)
Supplement: S2 Text — (DOCX) [file pone.0167983.s004.docx]

S2 Text. CrowdFlower job questions

Question 1: Is there mention of a specific place or time?

(Yes/No)

## Question 2: Does the narrator refer to himself in the text? Look for pronouns such as we, our, I, and my.

(Yes/No)

Question 3: Count the number of times that sensory or emotional language is used. Look for words that represent sight, sound, smell, taste, touch, or feelings.

(Select a number)

Question 4: Count the number of times that conjunctions are used in the text. Look for words or phrases that signify cause and effect (e.g. consequently; therefore; as a result; so; for this reason), contrast (e.g. however; although; surprisingly), or temporal ordering (e.g. then; next; first; second).

(Select a number)

Question 5: Count the number of times that words or phrases from one sentence are used to create an explicit link to the sentence immediately before it (e.g. these benefits; this finding; or 'costs...the costs are...'; 'trend...this trend indicates...'; 'profit...a significant contribution to profit...').

(Select a number

Tip: Look for logical linkage between ideas. Words or phrases that create explicit links include either a specific reference back to the same thing or repetition of a word from the previous sentence, provided it carries the same meaning.

## Question 6: Does the text make an explicit appeal to the reader or a clear recommendation of action (e.g. We hope conservation professionals value the potential of...)?

(Yes/No)
