# Supplementary figures and images for: Narrative Style Influences Citation Frequency in Climate Change Science
Source: PLoS One. 2016 Dec 15;11(12):e0167983. doi: 10.1371/journal.pone.0167983 (PMC5158318; doi:10.1371/journal.pone.0167983)

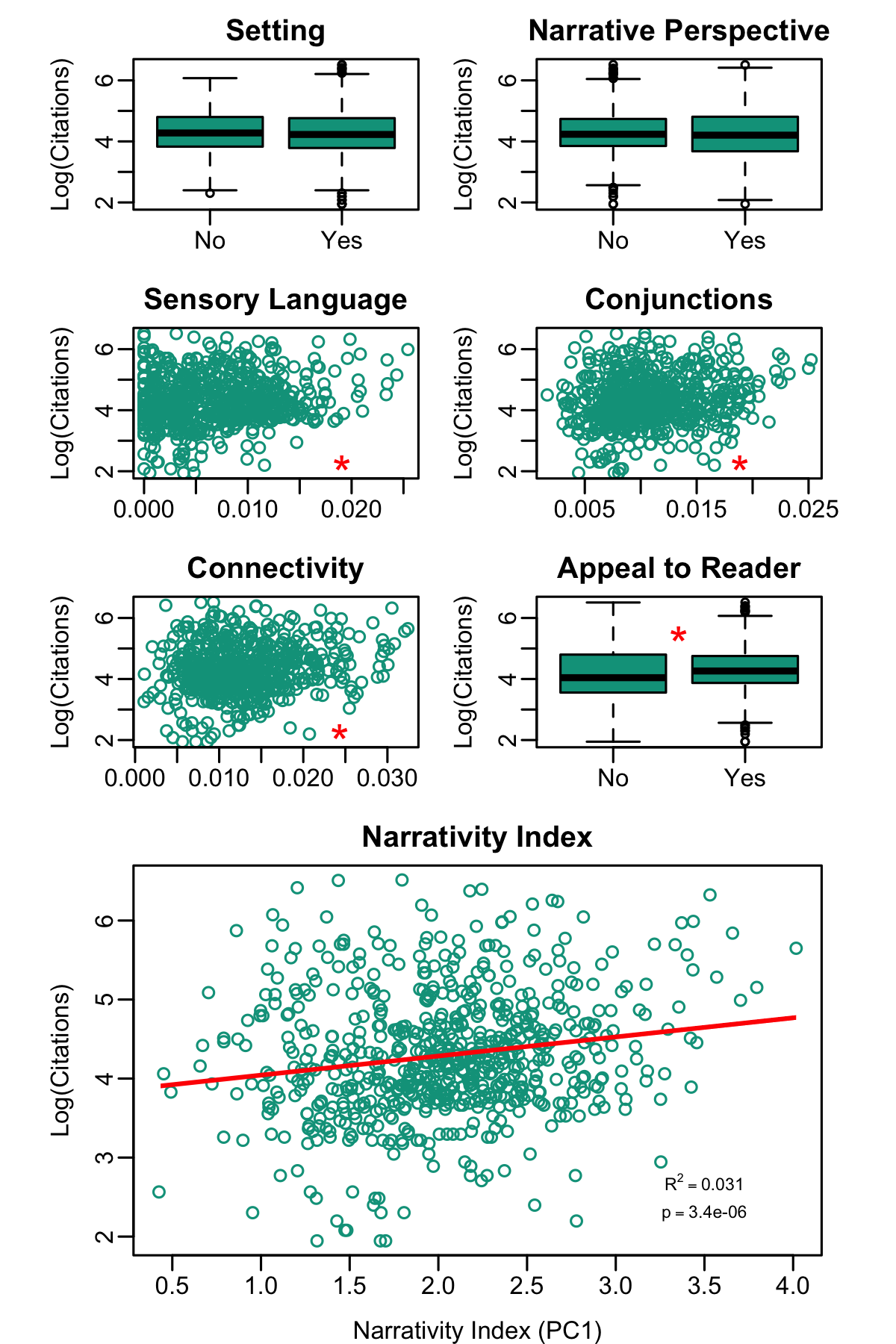

Supplement: S1 Fig — We identified outlier abstracts by fitting appropriate probability distributions to the non-binary independent variables (“conjunctions” (per abstract word), “connectivity” (per abstract word), log(abstract length), log(number of authors); gamma, gamma, normal, and gamma distributions, respectively) and to the dependent variable (log(citations); normal), and excluding responses with a likelihood < 0.01. Consequently, abstracts with very large or very small numbers of conjunctions or connective phrases—or extreme values for word count, number of authors, or number of citations—were removed from the dataset. In total, 46 outliers were removed from the dataset. This figure shows the results of the analyses described in the main paper, but carried out on this dataset with the 46 outlier abstracts removed. (TIFF) [file pone.0167983.s005.tiff]

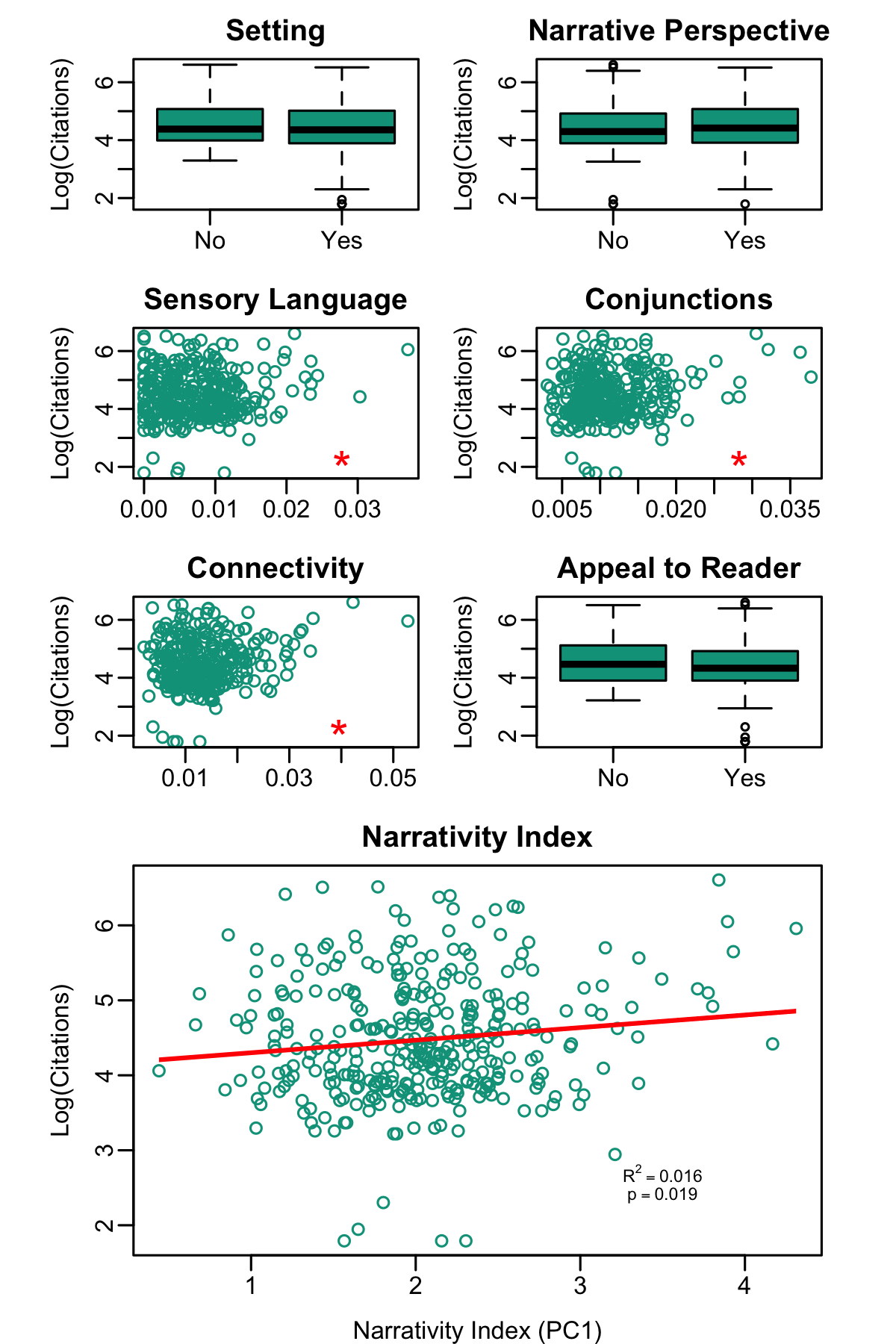

Supplement: S2 Fig — (TIFF) [file pone.0167983.s006.tiff]

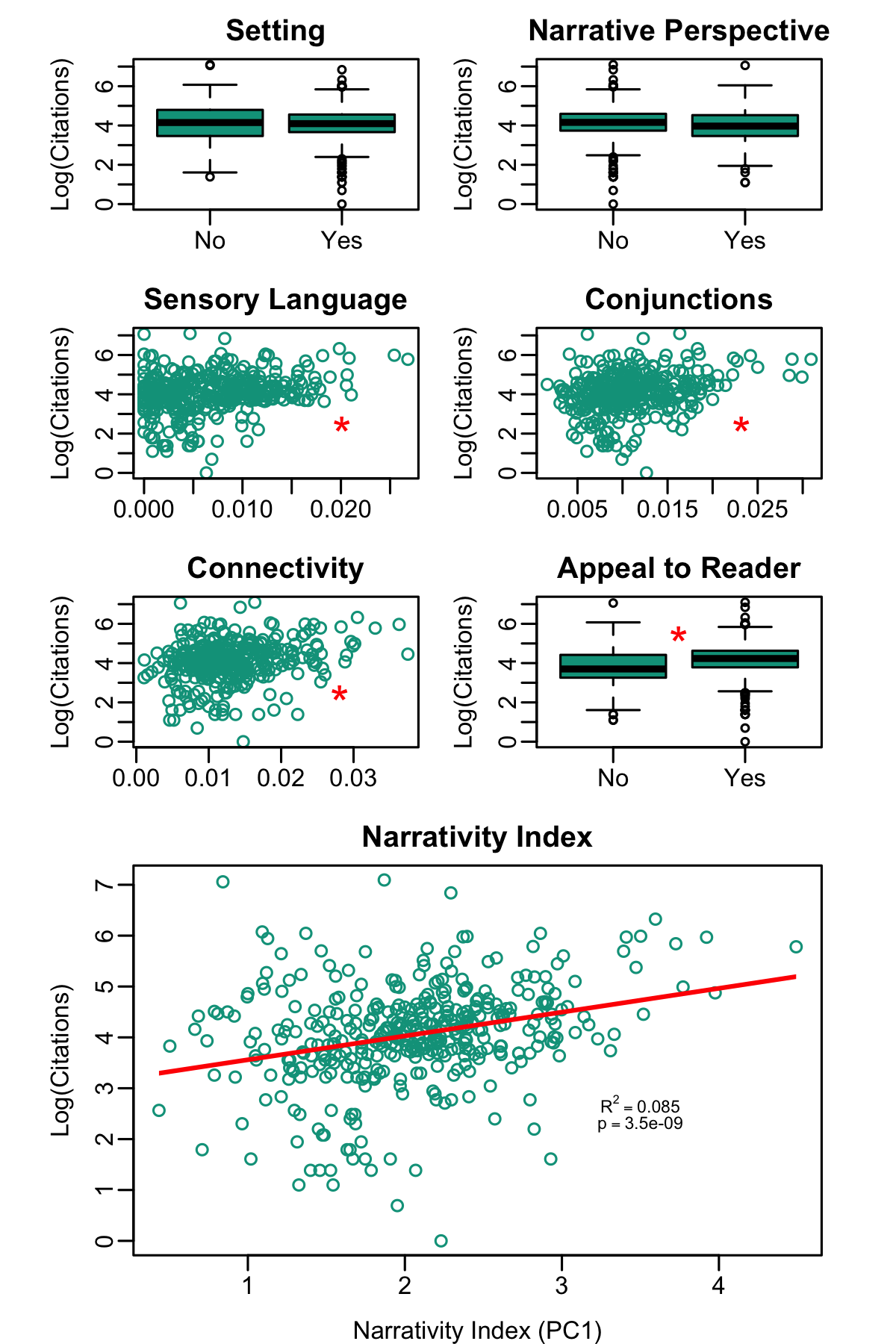

Supplement: S3 Fig — (TIFF) [file pone.0167983.s007.tiff]
